# Red LED light accelerates capsanthin accumulation and fruit ripening in pre-harvest pepper (*Capsicum annuum* L.)

**DOI:** 10.3389/fpls.2025.1680730

**Published:** 2025-12-03

**Authors:** Pengpeng Mao, Qi Yang, Tao Yang, Ping Zou, Yinjian Zheng

**Affiliations:** 1 College of Smart Agriculture (Research Institute), Xinjiang University, Urumqi, China; 2 College of Agriculture, Nanjing Agricultural University, Nanjing, China; 3 College of Agronomy and Biotechnology, Southwest University, Chongqing, China; 4 Institute of Agricultural Mechanization, Xinjiang Academy of Agricultural Sciences, Urumqi, China

**Keywords:** red LED light, fruit ripening, capsanthin, pepper, pre-harvest regulation, color change, virus-induced gene silencing

## Abstract

As an essential environmental regulator, light plays a crucial role in pre-harvest fruit quality and fruit ripening. However, developing efficient light-based strategies for precise regulation during critical crop growth stages remains a substantial challenge in controlled-environment agriculture. Fruit ripening in pepper (*Capsicum annuum* L.) is characterized by the development of its distinctive red color, which is primarily driven by the accumulation of capsanthin, a carotenoid that serves as the principal pigment in ripe pepper fruits. Nevertheless, the mechanism by which red LED light regulates capsanthin accumulation, and thus the color change of pepper fruits, remains unclear. In this study, ‘Zunla-1’ pepper fruits at the green ripening stage (30 days after flowering, DAF) were selected for pre-harvest red LED light treatment. Results showed that red LED light significantly promoted the color change of pepper fruits and accelerated capsanthin accumulation compared to white LED light. Additionally, red LED light upregulated the expression of carotenoid biosynthesis genes *PSY*, *LCYB*, *CRTZ*, and *CCS* at 34 and 38 DAF, with *CCS* exhibiting the highest expression at 38 DAF. Correspondingly, the concentrations of PSY, LCYB, CRTZ, and CCS enzymes were significantly increased under red LED light from 34 to 46 DAF, indicating that red LED light accelerated capsanthin accumulation more rapidly than white LED light. Virus-induced gene silencing assays confirmed that *CCS* (*Capana06g000615*) exhibited a stronger response to red LED light than *PSY* (*Capana04g002519*), *LCYB* (*Capana05g000023*), and *CRTZ* (*Capana03g002170*). Collectively, these findings demonstrate that pre-harvest red LED light accelerates pepper fruit ripening and capsanthin accumulation, and *CCS* plays a pivotal role in the red LED light-mediated regulatory process. This study provides novel insights into light-regulated fruit ripening and presents potential strategies for optimizing vegetable quality through targeted photobiological interventions.

## 1 Introduction

Pepper (*Capsicum annuum* L.), a widely cultivated vegetable crop in the *Solanaceae* family, is recognized for its diverse fruit colors (e.g., green, red, orange, purple) that greatly enhance its appeal to consumers (De Sá Mendes and Branco De Andrade Gonçalves, 2020; Liu et al., 2022a; Jiménez-Viveros et al., 2023). Additionally, pepper fruits are rich in health-promoting compounds, such as carotenoids, phenols, flavonoids, and capsaicinoids, which also have considerable value across various industries (De Sá Mendes and Branco De Andrade Gonçalves, 2020; Darko et al., 2022; Rodrigues-Salvador et al., 2022). However, in open-field cultivation environments, pepper plants are highly susceptible to abiotic stresses, including drought, high temperatures, and excessive light, which cause issues like fruit cracking, deformities, and yield loss (Espley and Jaakola, 2023). In modern pepper production systems, the use of supplemental lighting and light-emitting diodes (LEDs) by greenhouses and plant factories has emerged as a critical strategy to alleviate these challenges and enhance food security (Poonia et al., 2022; Thilini Deepashika Perera et al., 2022). Thus, to optimize pepper cultivation and improve pepper fruit quality, it would be particularly valuable to clarify the effects of LED light on the color change and metabolic shifts, particularly during the critical stages of fruit ripening and phytochemical biosynthesis.

Carotenoids are involved in photosynthesis, inhibit the accumulation of reactive oxygen species (ROS), and facilitate the synthesis of other essential phytochemicals (Pérez-Ambrocio et al., 2018; De Sá Mendes and Branco De Andrade Gonçalves, 2020; Villa-Rivera et al., 2022). They also specifically act as potent antioxidants and play a crucial role in isoprenoid biosynthesis in plants (Yoo et al., 2019). Based on their chemical structure, carotenoids are categorized into two main groups: hydrocarbon-type carotenoids (carotenes) and oxygen-containing carotenoids (xanthophylls) (Castillejo et al., 2022). The carotenoid biosynthesis pathway begins with phytoene synthase (PSY), which catalyzes the production of 15-cis-phytoene and C5 isopentenyl diphosphate (IPP); subsequently, phytoene desaturase (PDS), ξ-carotene desaturase (Z-ISO), ξ-carotene isomerase (ZDS), and carotenoid isomerase (CRTISO) mediate desaturation and isomerization reactions to form all-trans-lycopene (Ma et al., 2021; Villa-Rivera et al., 2022). Furthermore, ϵ-ring (LCYE) and β-ring (LCYB) cyclic end cyclase separate all-trans-lycopene into two branches, producing orange α-carotene and β-carotene, respectively. Finally, under the catalysis of zeaxanthin epoxidase (ZEP) and beta-carotene hydroxylase (CRTZ), β-carotene is converted into antheraxanthin. Capsanthin, the most abundant carotenoid in pepper fruits, is synthesized by capsanthin-capsorubin synthase (CCS) (Kilcrease et al., 2015) and contributes to the characteristic red color of pepper fruits as they ripen, a process regulated by phytohormones, dynamic gene expression, metabolic rewiring, and changes in enzyme activity (Romanazzi et al., 2016; Nie et al., 2023; Xu et al., 2024). However, the regulatory effects of abiotic factors on carotenoid biosynthesis and the fruit coloration process remain insufficiently elucidated.

Fruit ripening is an orchestrated physiological and biochemical cascade characterized by sugar accumulation, reduced organic acid content, dynamic changes in water status and texture, and intricate shifts in pigment composition (De Sá Mendes and Branco De Andrade Gonçalves, 2020; Rodrigues-Salvador et al., 2022; Thilini Deepashika Perera et al., 2022). Controlled acceleration of this process, known as pre-harvest ripening, serves as an important strategy to enhance both market supply efficiency and the sensory/nutritional quality of fruits, thereby enhancing agricultural yields (Liu et al., 2022b). Its value is further heightened in scenarios that demand rapid responses to consumer needs, mitigation of economic losses, and safeguarding of food security—particularly amid global challenges such as economic volatility, natural disasters, and population-driven food scarcity (Espley and Jaakola, 2023). Conventional strategies for regulating pre-harvest ripening have traditionally utilized three primary approaches: ethylene fumigation, precise temperature and humidity control, and exogenous phytohormone application (Ilić and Fallik, 2017). However, these well-established techniques necessitate the meticulous calibration of gas concentrations, treatment durations, and environmental parameters, among other factors. Such technical complexities create substantial barriers to their scalable adoption in large-scale commercial fruit production, emphasizing the need for more feasible and efficient strategies for regulating ripening.

LED light has emerged as a prevalent tool for regulating the light environment in plant cultivation (Yoo et al., 2019). Studies have demonstrated that LED light can extend the shelf life of horticultural products and enhance their post-harvest quality (Liu et al., 2022a; Thilini Deepashika Perera et al., 2022; Martínez-Zamora et al., 2023). Additionally, LED light can be used to regulate the synthesis and metabolism of various phytochemicals, as has been documented in crops that include tomato, pak choi, and lettuce (Poonia et al., 2022; Yang et al., 2023; Mao et al., 2024). However, continuous LED light exposure across all plant growth stages can drive high electricity costs, which imposes a significant economic burden on plant factories and vertical farming systems. Thus, optimizing LED light utilization during critical growth stages (e.g., color change and quality formation) is essential for conserving energy, promoting plant growth, and improving produce quality (Kaiser et al., 2024). Red LED light, an essential component of the light spectrum, regulates various plant growth processes, including seed germination, morphogenesis, flowering, and fruiting (Pattison et al., 2018; Yoo et al., 2019; Zhang et al., 2022; Nie et al., 2023). It has also been reported that red LED light accelerates post-harvest color change of pepper fruits (Pola et al., 2020a) and modulates pepper branching to support high-quality fruit development (Nie et al., 2023). Nevertheless, the effects of pre-harvest red LED light on color change and capsanthin accumulation in pepper fruits remain insufficiently investigated.

This study aimed to elucidate the impact of pre-harvest red LED light in regulating capsanthin accumulation and its effect on color change in *Capsicum annuum* L. cv. ‘Zunla-1’ fruits at the green ripening stage (30 days after flowering, DAF). Pepper fruits were subjected to red LED light treatment for 4, 8, and 16 days, corresponding to sampling at 34, 38, and 46 DAF, respectively. Color changes, capsanthin content, and the expression levels of genes involved in capsanthin metabolism under red LED light were compared with those of fruits exposed to white LED light. To further clarify the mechanism by which red LED light regulates capsanthin metabolism, virus-induced gene silencing (VIGS) was employed to silence four key genes involved in the carotenoid biosynthesis pathway (namely, *PSY*, *LCYB*, *CRTZ*, and *CCS*). Red LED light significantly promoted the color change of fruit and enhanced capsanthin accumulation compared to white LED light. These effects were associated with the red light signaling-mediated upregulation of genes involved in capsanthin metabolism. Correspondingly, the concentrations of enzymes encoded by *PSY*, *LCYB*, *CRTZ*, and *CCS* were significantly higher under red LED light compared with those under white LED light, indicating that red LED light accelerates capsanthin accumulation. Furthermore, VIGS assays revealed that *CCS* plays a pivotal role in regulating color change and capsanthin accumulation in pepper fruits. This study provides important insights into light-mediated regulation of fruit quality and offers an empirical foundation for further molecular investigations into red light-regulated capsanthin metabolism in pepper and perhaps related crops.

## 2 Materials and methods

### 2.1 Seedling cultivation and plant growth

This study was performed at the College of Agronomy and Biotechnology, Southwest University (Chongqing, China). Seeds of pepper (*Capsicum annuum* L. cv. Zunla-1) were soaked in ultrapure water for 2 h, followed by treatment with a 1.0% cupric sulfate solution for 10 min and subsequent treatment with a 0.3% zinc sulfate solution for 15 min. After being thoroughly rinsed with ultrapure water, the seeds were soaked in 55 °C water for 15 min and then in room-temperature ultrapure water for 8 h. Hydrated seeds were germinated in an incubator on damp towels at 28 °C for 8 days. Germinated seeds were transplanted into 32-well seedling trays filled with a growing medium consisting of peat, vermiculite, and perlite (v:v:v, 3:1:1) and cultivated under white light in an artificial climate chamber. The chamber conditions were maintained as follows: photosynthetic photon flux density (PPFD) of 200 μmol m^−2^ s^−1^ (SS-110, Apogee Instruments, Logan, UT, USA), 12-h photoperiod, 25 °C temperature, and 80% relative humidity. Seedlings were fertilized weekly with half-strength Hoagland nutrient solution (pH approximately 6.0) to support healthy growth. Once seedlings had developed 10 uniform-sized true leaves, they were transplanted into pots (20 cm in height, 18 cm in diameter) and thereafter cultivated in a climate-controlled greenhouse under conditions consistent with those of the artificial climate chamber. Fifty days post-transplantation, fruits from plants with the same flowering period were labeled and subjected to pre-harvest LED light treatments at the green ripening stage (30 days after flowering, DAF).

### 2.2 Pre-harvest light treatments and sampling

For pre-harvest light treatments, white and red LED lights (T8 integration tube, Tonghuo Technology Co., Ltd., Xiamen, China) were employed as light sources. Fifteen pepper plants were evaluated for each treatment duration, namely 4, 8, and 16 days, to capture key stages of fruit development. The PPFD at the canopy level was maintained at 140 μmol m^−2^ s^−1^ under a 12-h photoperiod. The LED spectra and detailed treatment conditions are illustrated in **Supplementary Figure S1**. Ten fruits were harvested at 0, 4, 8, and 16 days (30, 34, 38, and 46 DAF, respectively) after treatment, respectively. Pepper samples were collected after removing seeds and placental tissue, and they were rapidly frozen in liquid nitrogen and stored at -80 °C until analysis. Each light treatment was conducted across three biological replicates.

For the VIGS experiments, four key genes in the capsanthin biosynthesis pathway—*PSY* (*Capana04g002519*), *LCYB* (*Capana05g000023*), *CRTZ* (*Capana03g002170*), and *CCS* (*Capana06g000615*)—were silenced using TRV-induced VIGS in labeled fruits at 30 DAF. For each gene silencing assay, eight pepper plants were used, with 16 fruits injected per plant. The injected fruits on these plants were first incubated in the dark at 25 °C and under 80% relative humidity for 2 days and then transferred to an artificial climate chamber and exposed to red LED light (peak wavelength, 660 nm) for 16 days with a PPFD of 140 μmol m^−2^ s^−1^ and a 12-h photoperiod. After 16 days of red LED light exposure, the capsanthin content, fruit color changes, and relative expression of key genes in the silenced fruit samples were analyzed. Two control groups consisted of untreated wild-type (WT) fruits and fruits injected with the empty TRV vector (TRV/00). The VIGS experiment was performed across three biological replicates.

### 2.3 Analysis of fruit surface color

The skin color of pepper fruits was assessed using a colorimeter (3nh, Colorimeter CR2; Beijing Moleck Biological Technology Co., Ltd., Beijing, China). The L*, a*, and b* color space parameters were recorded and analyzed. The hue angle (h°, in degrees) was calculated according to the following equations: h° = tan^–1^(b^*^/a^*^) when a^*^ > 0, and h° = 180° + tan^–1^(b^*^/a^*^) when a^*^ < 0. Color saturation was computed as color saturation = (a^2^ + b^2^)^0.5^, and the color index was calculated as color index = (1000 × a^*^)/(L^*^ × b^*^). Hue angle was used as a key indicator of fruit color, such that higher values indicate greener fruits and lower values indicate redder fruits. For each treatment, three biological replicates were evaluated, with each replicate containing five fruits. Each fruit was measured at six distinct spatial points on its surface to account for color uniformity. The reported color parameters for each treatment represent the mean value derived from all these measurements.

### 2.4 Determination of capsanthin content

Capsanthin content was determined according to a slightly modified version of the method described by Kulkarni et al. (2020). For each treatment, lyophilized pepper pericarp samples (1.00 g each) were separately weighed and added to containers containing acetone. The mixtures were vigorously shaken for 4 h, then diluted with acetone to a final volume of 250 mL, and incubated at room temperature for 10 min to allow residues to settle. Next, 10 mL of the supernatant was carefully pipetted from each container and further diluted to 100 mL with acetone. After thorough mixing by shaking, the absorbance of each diluted solution was measured at 460 nm using acetone as the blank control. Standard solutions of capsanthin were prepared in acetone using pure capsanthin in order to generate a standard curve. Capsanthin content in each sample was calculated based on the standard curve and expressed as micrograms per gram of dry weight (μg g^−1^ DW).

### 2.5 Measurement of American Spice Trade Association color value

The American Spice Trade Association (ASTA) color value was determined using the method described by Ko et al. (2022). For each sample, freeze-dried pepper powder (100 mg) was extracted with 100 mL of acetone and incubated in the dark at room temperature for 16 h. Following sample extraction, the mixtures were clarified by filtration using qualitative filter paper to separate solid residues, and the supernatants were collected. The absorbance of each supernatant was measured at 460 nm using acetone as the blank control. The ASTA color value was calculated using the formula ASTA color value = (absorbance at 460 nm × 16.4 × *If*)/sample weight, where *If* is the instrument correction factor (calibrated using a glass reference standard).

### 2.6 Total protein extraction and key enzyme concentration measurement

Total protein extraction from pepper fruits was performed using the Plant Protein Extraction Kit (No. C500053, Sangon Biotech Co., Ltd., Shanghai, China) according to the manufacturer’s instructions. Briefly, 0.10 g of pepper fruit tissue that had been ground into powder in liquid nitrogen was mixed with 1 mL of Solution A and 0.7 μL of Solution C. After resuspension by shaking, the mixture was incubated at -20 °C for 45 min. Following centrifugation at 15,000 rpm for 15 min at 4 °C, the resulting precipitate was collected. The precipitate was resuspended in 1 mL of Solution B, 10 μL of Solution D, and 0.7 μL of Solution C and then incubated at -20 °C for 60 min. After a second centrifugation at 15,000 rpm for 15 min at 4 °C, the precipitate was again treated with 1 mL of Solution B, 10 μL of Solution D, and 0.7 μL of Solution C. The resuspended mixture was immediately centrifuged at 15,000 rpm for 15 min at 4 °C again. The final precipitate was dissolved in 0.5 mL of buffer, and after centrifugation at 15,000 rpm for 5 min at 4 °C once again, the supernatant was collected as the total protein extract sample.

The concentrations of PSY, LCYB, CRTZ, and CCS enzymes were determined using ELISA kits (Bioroyee Biotechnology Co., Ltd, Beijing, China) following the corresponding protocols provided by the manufacturer. Standard solutions for PSY, LCYB, CRTZ, and CCS were first diluted individually. Fifty microliters of each standard solution was added to the corresponding wells of a pre-coated ELISA plate; at the same time, 40 μL of sample diluent and 10 μL of the test protein extract sample were mixed thoroughly, and this mixture was added to separate wells. Each plate was sealed with a film and incubated at 37 °C for 30 min. After discarding the liquid, wells were filled with washing buffer (diluted with ddH_2_O) for 30 s, and then, the buffer solutions were discarded; this washing step was repeated five times. After patting each plate dry, 50 μL of enzyme conjugate reagent was added to both standard and sample wells, followed by incubation at 37 °C for 30 min. The washing procedure was repeated five times as described above. Subsequently, 50 μL of chromogen solutions A and B were added to each well, mixed thoroughly, and incubated in the dark at 37 °C for 10 min. The reaction was terminated by adding 50 μL of stop solution to each well. Absorbance was measured at 450 nm using a Spark multifunctional microplate reader (Tecan, Männedorf, Switzerland), with the blank well used for zero calibration. Finally, the concentrations of PSY, LCYB, CRTZ, and CCS enzymes in the test samples were calculated based on their respective standard curves.

### 2.7 Total RNA extraction and RT-qPCR assays

Total RNA was isolated from pepper fruit tissues using the RNA Easy Fast Plant Tissue Kit (No. DP452, TIANGEN Biotech Co., Ltd., Beijing, China) according to the manufacturer’s protocol. The quantity and quality of extracted RNA were assessed using a NanoPhotometer spectrophotometer (Shimadzu Corporation, Kyoto, Japan), and cDNA was synthesized from the quality-checked RNA using the FastKing RT Kit (with gDNase) (TIANGEN Biotech Co., Ltd., Beijing, China) following the manufacturer’s protocol. RT-qPCR was performed using the Talent qPCR PreMix (SYBR Green) Kit, and amplification was conducted under the following thermal cycling program: initial denaturation at 95 °C for 2 min, followed by 40 cycles at 95 °C for 5 s, 60 °C for 10 s, and extension at 72 °C for 15 s. Each reaction volume (20 μL) contained 1 μL of cDNA template, 0.6 μL of each primer (10 μM), 10 μL of 2×FastReal qPCR PreMix, 2 μL of 50×ROX Reference Dye, and 5.8 μL of RNase-Free ddH_2_O. All RT-qPCR experiments were performed across three biological replicates, and transcript abundance values represent the average of three technical replicates per biological replicate. The pepper *UBI3* gene was used as the internal reference gene to normalize gene expression levels, and relative expression was calculated using the 2^-ΔΔCT^ method (Tian et al., 2014; Pola et al., 2020b). The RT-qPCR primer sequences are listed in **Supplementary Table S1**.

### 2.8 Virus-induced gene silencing assays

VIGS assays were performed using pTRV1 and pTRV2 vectors (Miaoling Biological Technology Co., Ltd., Wuhan, China). The coding sequences of the *PSY*, *LCYB*, *CRTZ*, and *CCS* genes were individually cloned into the pTRV2 vector, which contains *BamH* I and *Kpn* I restriction enzyme sites (**Supplementary Table S2**). The resulting recombinant plasmids pTRV2/*PSY*, pTRV2/*LCYB*, pTRV2/*CRTZ*, and pTRV2/*CCS* were transformed into chemically competent *E. coli* DH5α cells. Positive clones were screened by colony PCR and verified by Sanger sequencing. Subsequently, the validated recombinant plasmids were transformed into *Agrobacterium* strain GV3101.

*Agrobacterium* colonies carrying the pTRV2 recombinant vectors were grown at 28 °C in Luria-Bertani medium containing 50 mg/L rifampicin and 50 mg/L kanamycin until the optical density at 600 nm (OD_600_) reached 0.5–0.8. After centrifugation, the cells were resuspended in MES buffer (150 mM acetylcinnamaldehyde, 10 mM MgCl_2_, 10 mM MES, pH 5.6) to an OD_600_ of approximately 0.6 and incubated at room temperature for 5 h. Prior to injection, equal volume aliquots of *Agrobacterium* culture carrying pTRV1 and pTRV2 (pTRV2/*PSY*, pTRV2/*LCYB*, pTRV2/*CRTZ*, and pTRV2/*CCS*) were mixed. Using a 1 mL syringe with a needle, the mixture was injected into the peel of pepper fruits at a volume of approximately 0.5 mL per fruit. The injected fruits were incubated in the dark at 25 °C and 80% relative humidity for 2 days and then exposed to red LED light (660 nm) for 16 days in an artificial climate chamber with a PPFD of 140 μmol m^−2^ s^−1^ and a 12-h photoperiod.

### 2.9 Statistical analysis

All experiments included three biological replicates. Data were analyzed using SPSS Statistics 25.0 software (IBM Corp., Armonk, NY, USA). Statistically significant differences among treatments were determined by one-way analysis of variance (ANOVA) followed by the least significant difference test, with a significance threshold of *p* < 0.05. Figures were generated using OriginPro 10.0 software (OriginLab, Northampton, MA, USA).

## 3 Results

### 3.1 The positive effect of pre-harvest red LED light on the color phenotype of pepper fruits

**Figure 1:**
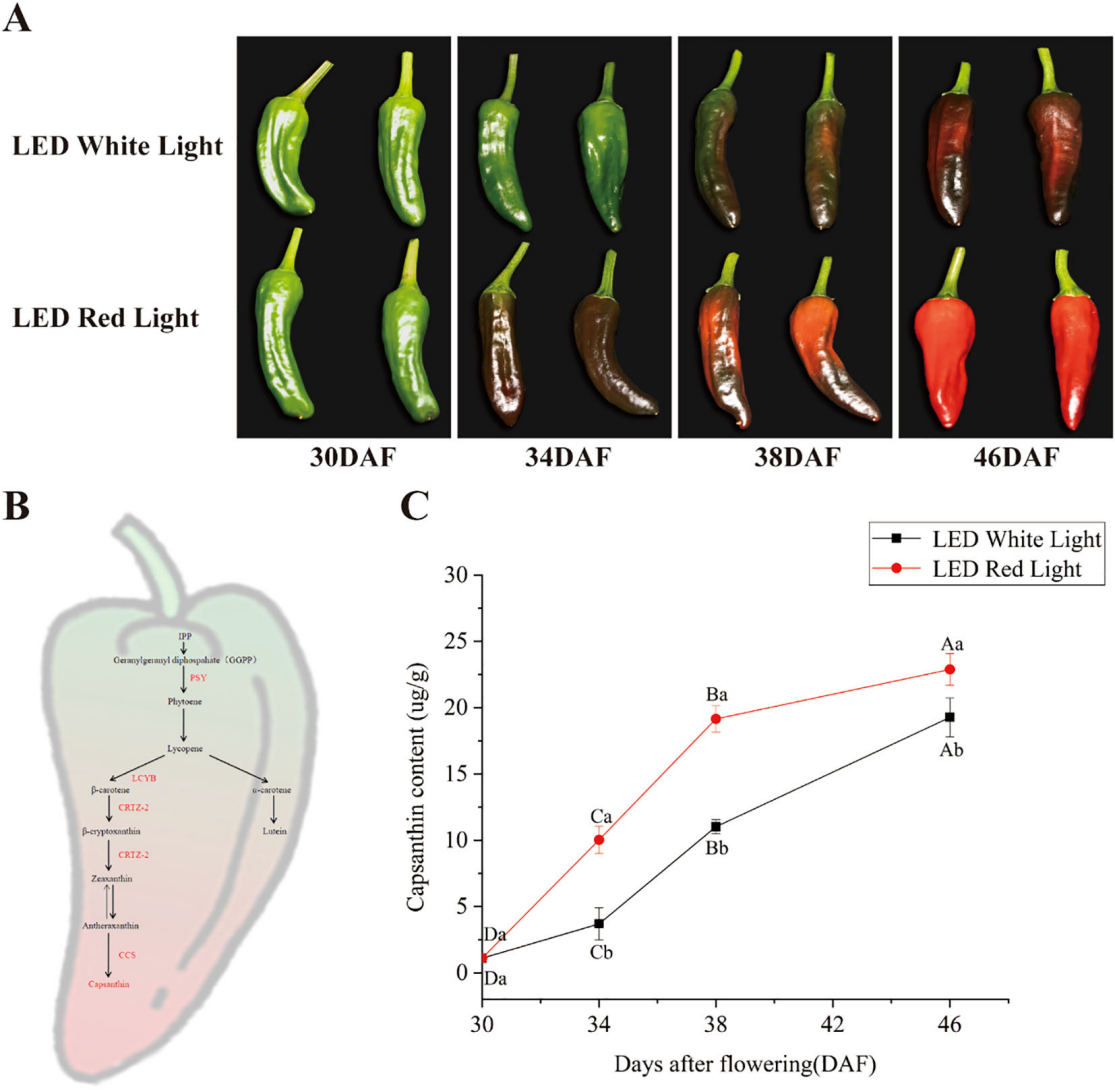
Effects of pre-harvest LED light on color change and capsanthin content in pepper fruits. **(A)** Color changes in pepper fruits under pre-harvest white and red LED light over different treatment durations. **(B)** Schematic diagram of color change and capsanthin metabolism in pepper fruit (Tian et al., 2014; Ma et al., 2023). **(C)** Capsanthin content in pepper fruits under pre-harvest white and red LED light over different treatment durations. DAF: days after flowering. The key enzymes involved in capsanthin metabolism are indicated by red text to the right of arrows. Different capital and lowercase letters indicate significant difference at the *p* < 0.05 level under the same light treatment and same treatment duration, respectively.

Pepper fruits at the green ripening stage (30 days after flowering, DAF) displayed a uniform green color (**Figure 1A**). Under pre-harvest white LED light, fruits maintained a green color until 34 DAF, underwent an initial transition to a purple color by 38 DAF, and developed a fully purple color by 46 DAF (**Figure 1A**, top row). In contrast, pre-harvest red LED light notably accelerated the normal fruit color change: fruits reached the purple stage at 34 DAF, transitioned to the red stage by 38 DAF, and attained a fully red ripened stage by 46 DAF (**Figure 1A**, bottom row). The transition from green to purple and subsequently to red occurred substantially faster under red LED light relative to white LED light, with the final red coloration appearing deeper and more intense under red LED light (**Figure 1A**). This accelerated color transition was tightly coupled to the temporal dynamics of capsanthin metabolism and accumulation (**Figures 1B, C**). As depicted in **Figure 1C**, capsanthin content increased in a time-dependent manner with fruit development under both light treatments. Notably, at each developmental stage evaluated, capsanthin levels were significantly higher under red LED light compared with white LED light (**Figure 1C**). For example, at 34 DAF, capsanthin content under red LED light was approximately three-fold higher than that under white LED light. These results demonstrate that pre-harvest red LED light not only accelerated the rate of color change but also promoted the time-dependent accumulation of capsanthin in pepper fruits, thereby accelerating their transition to the red ripened stage.

### 3.2 Effects of pre-harvest red LED light on the expression of key structural genes in the capsanthin biosynthesis pathway

**Figure 2:**
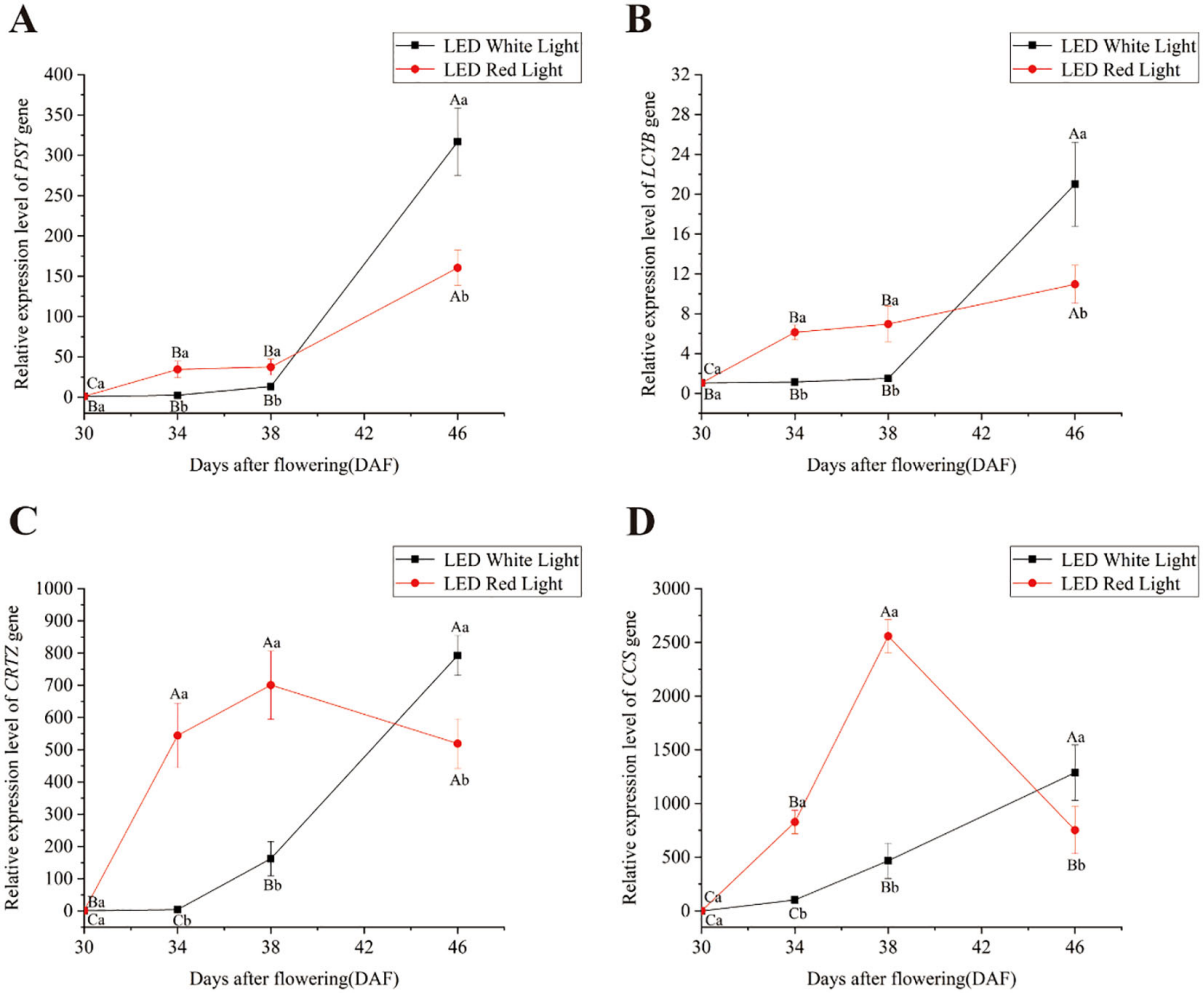
Effects of pre-harvest LED light on the relative expression levels of key structural genes in the capsanthin biosynthesis pathway. **(A)***PSY*; **(B)***LCYB*; **(C)***CRTZ*; **(D)***CCS*. Different capital and lowercase letters indicate significant difference at the *p* < 0.05 level under the same light treatment and same treatment duration, respectively.

To elucidate how pre-harvest red LED light regulates capsanthin biosynthesis, we analyzed the temporal expression dynamics of four key structural genes (*PSY*, *LCYB*, *CRTZ*, and *CCS*) in the capsanthin biosynthesis pathway across fruit developmental stages (30, 34, 38, and 46 DAF) under white and red LED light treatment (**Figure 2**). Under white LED light, the relative expression levels of *PSY* and *LCYB* exhibited no significant differences from 30 to 38 DAF (**Figures 2A, B**), while *CRTZ* and *CCS* also exhibited stable expression between 30 DAF and 34 DAF (**Figures 2C, D**). Notably, *PSY* and *LCYB* reached their maximum relative expression levels at 46 DAF under white LED light (**Figures 2A, B**), indicating late-stage activation of these genes in the capsanthin biosynthesis pathway had occurred. In contrast, red LED light treatment triggered more rapid and robust expression of all four key genes. Relative to the 30 DAF stage, the expression levels of *PSY*, *LCYB*, *CRTZ*, and *CCS* were significantly upregulated under red LED light. At 34 and 38 DAF, red LED light exposure exerted a stronger effect on the expression of these genes compared to white LED light, resulting in significantly higher expression levels of *PSY*, *LCYB*, *CRTZ*, and *CCS* (**Figure 2**). Notably, by 46 DAF, the relative expression levels of *PSY*, *LCYB*, *CRTZ*, and *CCS* were significantly lower under red LED light than under white LED light. Therefore, the capsanthin biosynthesis pathway was promoted under red LED light, with key structural genes exhibiting earlier expression under red LED light compared to white LED light.

### 3.3 Effects of pre-harvest red LED light on the concentration of key enzymes in the capsanthin biosynthesis pathway

**Figure 3:**
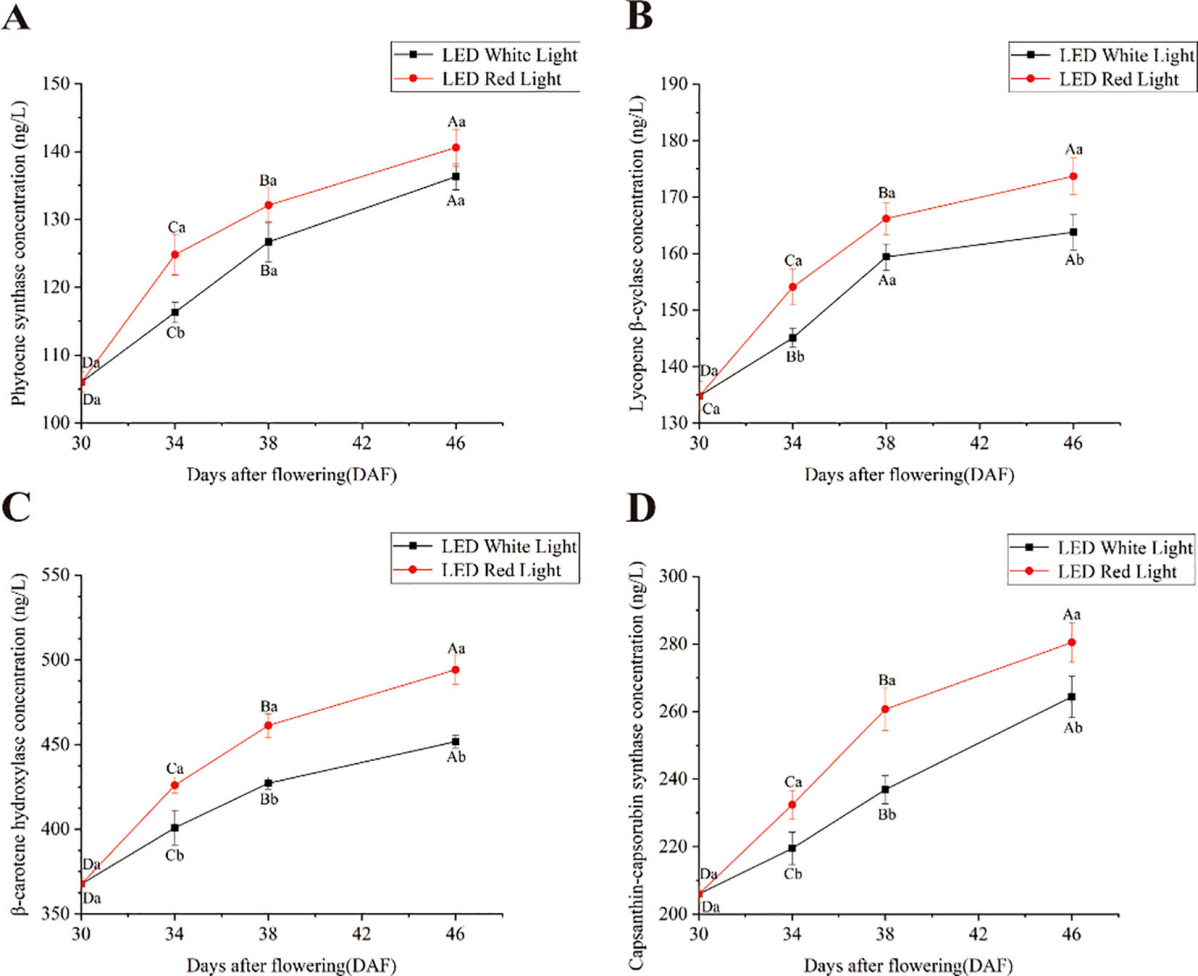
Effects of pre-harvest LED light on the concentration of key enzymes in the capsanthin biosynthesis pathway. **(A)** Phytoene synthase (PSY) concentration; **(B)** Lycopene β-cyclase (LCYB) concentration; **(C)** β-carotene hydroxylase (CRTZ) concentration; **(D)** Capsanthin-capsorubin synthase (CCS) concentration. Different capital and lowercase letters indicate significant difference at the *p* < 0.05 level under the same light treatment and same treatment duration, respectively.

The concentrations of key enzymes in the capsanthin biosynthesis pathway increased consistently from 30 to 46 DAF in pepper fruits, regardless of pre-harvest exposure to red or white LED light (**Figure 3**). However, at the same time points, the concentrations of phytoene synthase, lycopene β-cyclase, β-carotene hydroxylase, and capsanthin-capsorubin synthase were higher under red LED light than under white LED light. Despite this overall trend, no significant differences were observed in the concentration of phytoene synthase at 38 and 46 DAF or in the concentration of lycopene β-cyclase at 38 DAF between fruits under red and white LED light (**Figures 3A, B**). Collectively, these results indicated that pre-harvest red LED light increased the concentrations of key enzymes in the capsanthin biosynthesis pathway compared to white LED light.

### 3.4 Effect of silencing four key genes in the capsanthin biosynthesis pathway on fruit color and capsanthin content under red LED light

**Figure 4:**
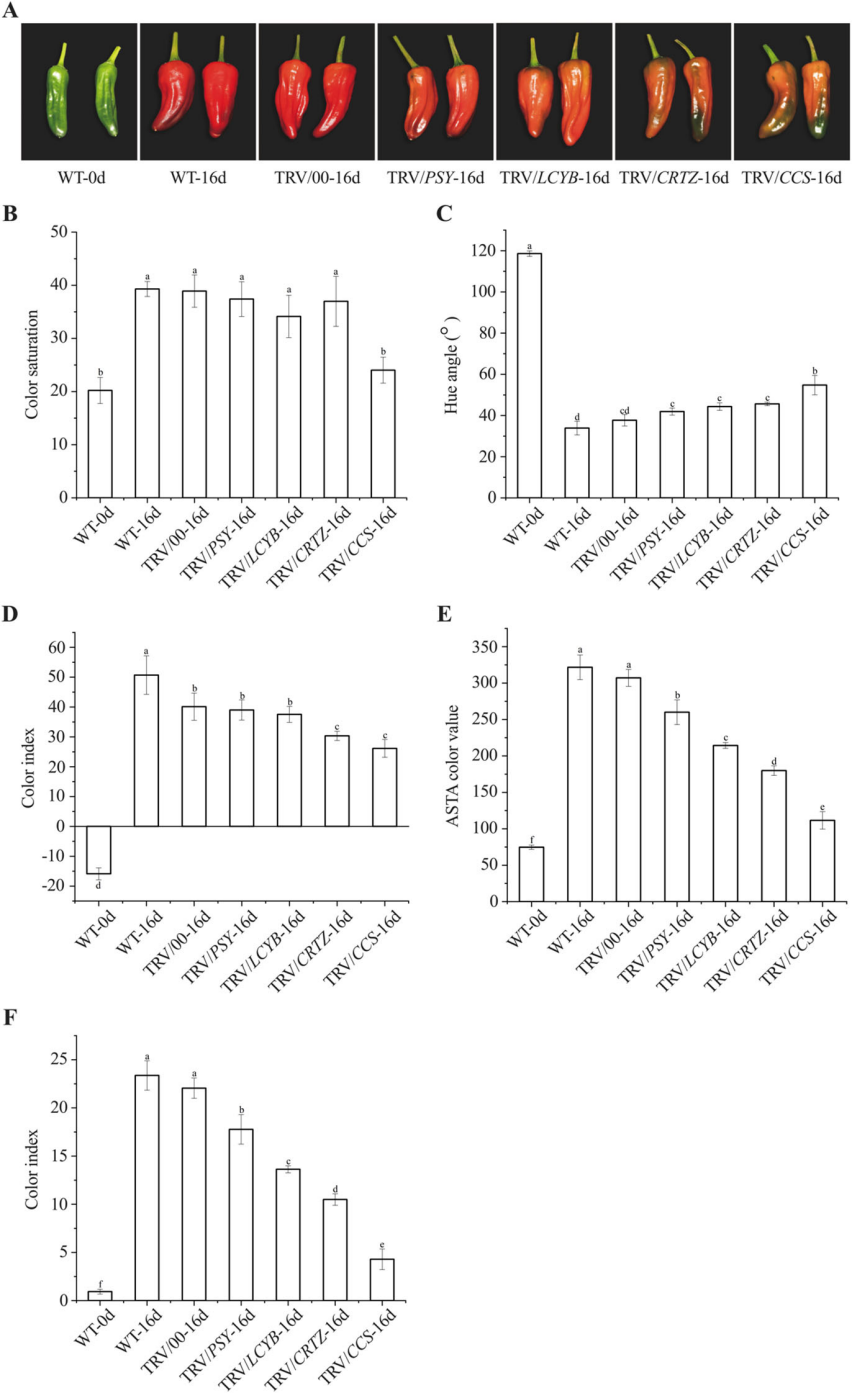
Effect of silencing four key genes in the capsanthin biosynthesis pathway on phenotypic changes and capsanthin content of pepper fruits under red LED light. **(A)** Color changes; **(B)** Color saturation; **(C)** Hue angle; **(D)** Color index; **(E)** ASTA color value; **(F)** Capsanthin content. Wild-type (WT) pepper fruits without injection prior to red LED light exposure are denoted as WT-0d; WT pepper fruits without injection after 16 days of red LED light exposure are denoted as WT-16d. Pepper fruits injected with the empty TRV vector after 16 days of red LED light exposure are denoted as TRV/00-16d; those injected with TRV/*PSY*, TRV/*LCYB*, TRV/*CRTZ*, and TRV/*CCS* after 16 days of red LED light exposure are denoted as TRV/*PSY*-16d, TRV/*LCYB*-16d, TRV/*CRTZ*-16d, and TRV/*CCS*-16d, respectively. Different letters above the bars indicate significant differences at *p* < 0.05.

At 30 DAF (WT-0d), pepper fruits still exhibited a green color phenotype. After 16 days of red LED light exposure (WT-16d), fruits injected with the empty TRV vector (TRV/00-16d) displayed a red color. In contrast, after the same red LED light treatment period, fruits injected with TRV/*PSY* or TRV/*LCYB* developed an orange-red color, whereas those injected with TRV/*CRTZ* or TRV/*CCS* remained green (**Figure 4A**). No significant differences in color saturation or hue angle were observed among TRV/*PSY*-16d, TRV/*LCYB*-16d, TRV/*CRTZ*-16d, and TRV/00-16d fruits. However, fruits in the TRV/*CCS*-16d group showed significantly lower color saturation and higher hue angle relative to the other groups (**Figures 4B, C**). Additionally, after 16 days of red LED light exposure, the color index of fruits in the TRV/*CRTZ*-16d group was significantly lower than that in the WT-0d and TRV/00-16d groups (**Figure 4D**). When compared to the WT-0d and TRV/00-16d groups, both ASTA color value and capsanthin content were significantly reduced in fruits with any of the four silenced genes (*PSY*, *LCYB*, *CRTZ*, and *CCS*), with the lowest values observed in the TRV/*CCS*-16d group (**Figures 4E, F**). These results suggest that silencing the four key genes in the capsanthin biosynthesis pathway significantly modulated the promoting effect of pre-harvest red LED light on capsanthin accumulation, with *CCS* exerting the most prominent regulatory effect.

### 3.5 Effect of silencing four key genes in the capsanthin biosynthesis pathway on their relative expression under red LED light

**Figure 5:**
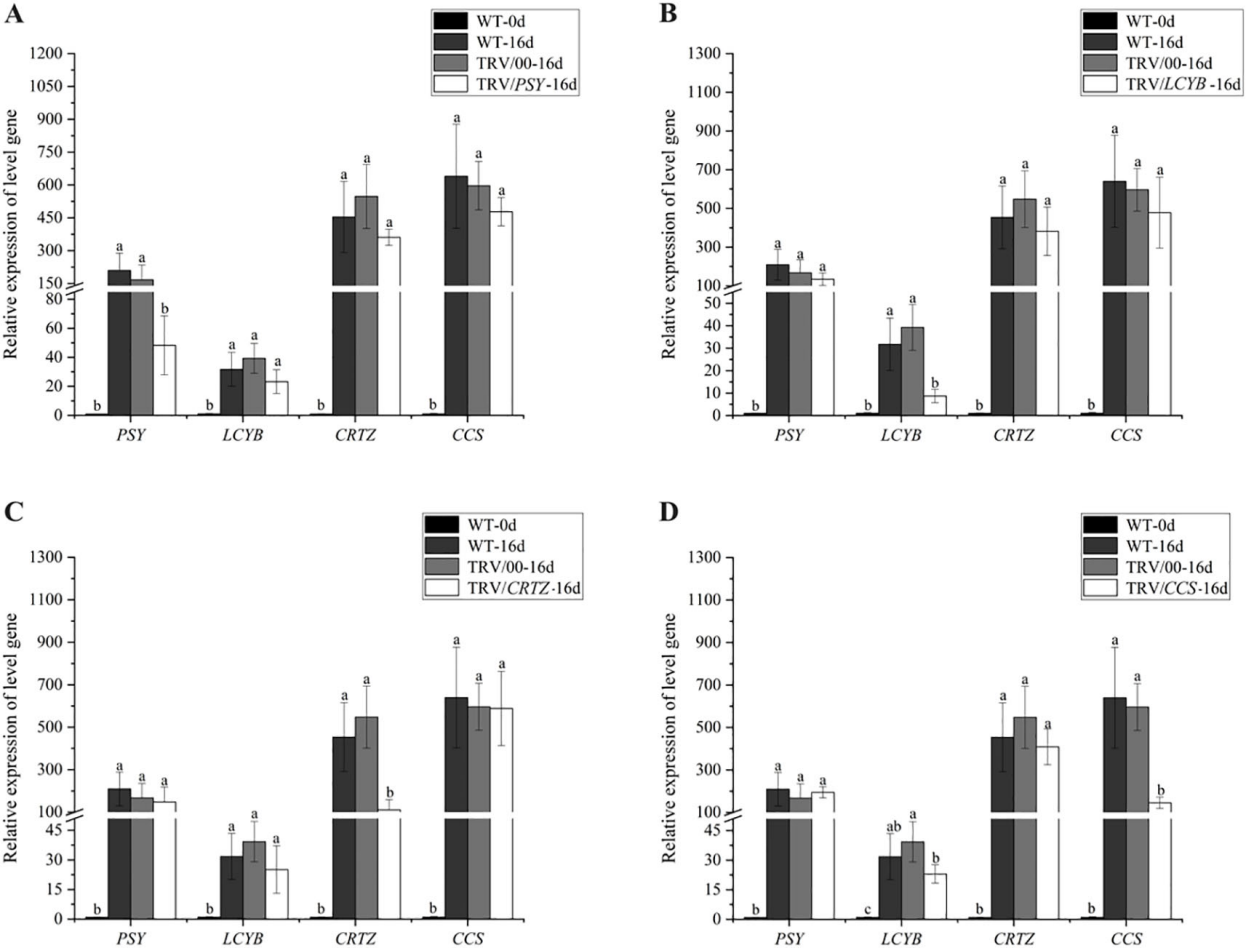
Effect of silencing four key genes in the capsanthin biosynthesis pathway on the relative expression of target genes in pepper fruits under red LED light was studied. **(A)***PSY*; **(B)***LCYB*; **(C)***CRTZ*; **(D)***CCS*. Different letters above the bars indicate significant differences at *p* < 0.05.

*PSY*, *LCYB*, *CRTZ*, and *CCS* are key genes in the capsanthin biosynthesis pathway. **Figure 5** shows how silencing these genes affects the relative expression levels of target genes in pepper fruits after 16 days of exposure to red LED light, compared with wild-type fruits at 0 days (WT-0d) and empty vector control fruits after 16 days under red LED light (TRV/00-16d). The relative expression of these genes was extremely low in WT-0d fruits, and no significant difference was observed between WT-16d and TRV/00-16d fruits. In contrast, after 16 days of red LED light exposure, the relative expression levels of *PSY*, *LCYB*, *CRTZ*, and *CCS* were significantly reduced in TRV/*PSY*-16d, TRV/*LCYB*-16d, TRV/*CRTZ*-16d, and TRV/*CCS*-16d fruits, respectively, compared to WT-16d and TRV/00-16d fruits. Notably, silencing just a single gene had only a minor impact on the relative expression of the other genes (**Figure 5**).

## 4 Discussion

Fruit ripening is a complex physiological and biochemical process involving sugar accumulation, organic acid reduction, and changes in water status, texture, and pigment composition (Yoo et al., 2019; De Sá Mendes and Branco De Andrade Gonçalves, 2020; Rodrigues-Salvador et al., 2022; Thilini Deepashika Perera et al., 2022). In recent years, LED light regulation has emerged as an efficient and sustainable approach for regulating pre-harvest ripening, with key advantages including its spectral precision and energy efficiency in improving fruit quality (Liu et al., 2022a; Espley and Jaakola, 2023; Chen et al., 2024). Pepper (*Capsicum annuum* L.), a globally important crop rich in health-promoting phytochemicals (e.g., capsanthin), is an ideal model for investigating light-mediated ripening mechanisms (Jiménez-Viveros et al., 2023). Notably, the ripening process of pepper fruits coincides with their capsanthin accumulation, making it a particularly suitable subject for systematic phenotypic, physiological, and molecular studies. Thus, understanding how LED light affects pre-harvest pepper fruit ripening and capsanthin accumulation is crucial for developing sustainable agricultural practices and addressing global food security challenges.

In the present study, pre-harvest red LED light (660 nm, 140 μmol m^−2^ s^−1^) significantly accelerated the color change of pepper fruits at the 30 DAF stage compared to white LED light: fruits treated with red LED light reached their fully red color stage by 46 DAF, whereas those treated with white LED light only developed a purple color (**Figure 1A**). Red light, an essential driver of photosynthesis, appears to promote pepper fruit ripening by boosting the accumulation of photosynthetic products. Previous studies have shown that higher proportions of blue light delay the ripening of purple pepper fruits (Liu et al., 2022b). This finding suggests that the delayed ripening of pepper fruits caused by white LED light in this study may be a consequence of its blue light content being higher than its red light content (**Supplementary Figure S1**). Notably, capsanthin content in pepper fruits at 38 DAF was higher under red LED light than that under white LED light (**Figure 1C**), confirming that red LED light not only accelerated pepper fruit ripening but also enhanced capsanthin accumulation. Furthermore, previous research has revealed that carotenoid biosynthesis genes, including *PSY*, *LCYB*, and *CCS*, display stage-specific expression during pepper fruit development, with most of these genes showing increased transcript abundance from 25 DAF onward (Liu et al., 2024). This is consistent with our finding that red LED light upregulates the expression of these genes at 34–38 DAF (**Figure 2**), suggesting that red light promotes the intrinsic developmental program governing carotenoid gene activation. In addition to its role in photosynthesis, red light is perceived by phytochromes, which convert the light signal into a regulatory signal for gene expression through transcription factors such as PIFs (phytochrome-interacting factors). In *Arabidopsis*, PIF1 directly binds to the *PSY* gene promoter to repress its expression, but this repression is alleviated when PIF1 is degraded in response to red light (Toledo-Ortiz et al., 2010). A similar mechanism operates in tomato fruit, where the PIF homologue PIF1a represses *PSY1* expression by binding to a PBE-box in its promoter (Llorente et al., 2016b). Although the promoters of pepper *PSY*, *LCYB*, *CRTZ*, and *CCS* have not been explicitly analyzed for PIF-binding sites in this study, the conservation of PIF-mediated regulation in tomato suggests a plausible mechanism for red light-induced carotenoid gene expression in pepper. Therefore, red light is a critical environmental factor that regulates pigment accumulation during fruit ripening. However, its effects on phytochemical content and fruit quality depend on factors including light dose, spectral composition, and crop variety (Espley and Jaakola, 2023; Jiménez-Viveros et al., 2023).

Relative expression analysis revealed that *PSY*, *LCYB*, *CRTZ*, and *CCS* genes were significantly upregulated under pre-harvest red LED light treatment for 4 and 8 days compared to white LED light treatment for the same time periods (**Figure 2**). Increased transcription of these key genes involved in capsanthin biosynthesis promotes their associated protein translation, thereby elevating the concentration of their corresponding enzymes and ultimately accelerating capsanthin accumulation (**Figure 3**). However, after 16 days of red LED light treatment, the relative expression levels of *PSY*, *LCYB*, *CRTZ*, and *CCS* were significantly lower than those under white LED light treatment. This observation suggests that the rate of capsanthin synthesis during pepper fruit ripening is initially rapid and then slows down (Liu et al., 2022b). At 38 DAF, most pepper fruits had turned red under red LED light, while very few fruits exhibited such a color change under white LED light. Additionally, plants possess feedback regulatory mechanisms that balance metabolite synthesis with normal growth, avoiding unnecessary resource waste and sustaining overall plant development (Li et al., 2023). Collectively, pre-harvest red LED light exposure for 16 days effectively accelerated pepper fruits ripening. However, further research is required to clarify the mechanism underlying the significant lower relative expression of *CCS* after 16 days of red LED light treatment compared to that at 8 days.

To further elucidate how red LED light regulates color change and capsanthin accumulation in pepper fruits, we performed VIGS assays targeting four key genes in the capsanthin biosynthesis pathway. VIGS is an effective method for targeted gene silencing, and its silencing effect persisted for 30 days in this study, which provide sufficient time to study the relationship between these genes and pepper fruit reddening under red LED light (Tian et al., 2014; Ma et al., 2023). As shown in **Figure 4A**, pepper fruits injected with TRV/*PSY* or TRV/*LCYB* displayed an orange-red color after 16 days of red LED light exposure, whereas those injected with TRV/*CRTZ* or TRV/*CCS* remained green. Additional indicators, including hue angle, color saturation, color index, ASTA color value, and capsanthin content, further confirmed the success of the VIGS assay and provided insights into the molecular mechanisms by which red LED light promotes fruit ripening (**Figure 4**). Compared to the control groups (WT-16d and TRV/00-16d), pepper fruits with silenced *PSY*, *LCYB*, *CRTZ*, or *CCS* exhibited significantly lower capsanthin content and failed to reach the red ripened stage after 16 days of red LED light treatment. Specifically, fruits with *CCS* silenced displayed a yellowish color rather than fully transitioning to green (Popovsky and Paran, 2000). The study used phenotypic and molecular analyses to reveal that *CCS* played the most prominent role in regulating capsanthin metabolism in response to red LED light (**Figure 5**). The pivotal role of *CCS* in red light-mediated capsanthin accumulation may be linked to its regulation by both light and developmental signals. In pepper, the MADS-RIN transcription factor directly binds to the *CCS* promoter and interacts with the R2R3-MYB transcription factor to synergistically activate its expression (Pereira and Teng, 2025). Since MADS-RIN is a key ripening regulator, its involvement suggests that red light may accelerate ripening partly through activating this module. Whether red light influences the stability or activity of PIF-like repressors in pepper, as observed in tomato (Llorente et al., 2016a), remains to be determined, but the conserved role of phytochromes in fleshy fruits supports the existence of a PIF-dependent pathway that modulates *PSY* and potentially *CCS* expression (Llorente et al., 2017). Future studies should focus on identifying more transcription factors that modulate capsanthin metabolism under red LED light, with *CCS* serving as a target gene in further investigations of the underlying molecular mechanisms.

**Figure 6:**
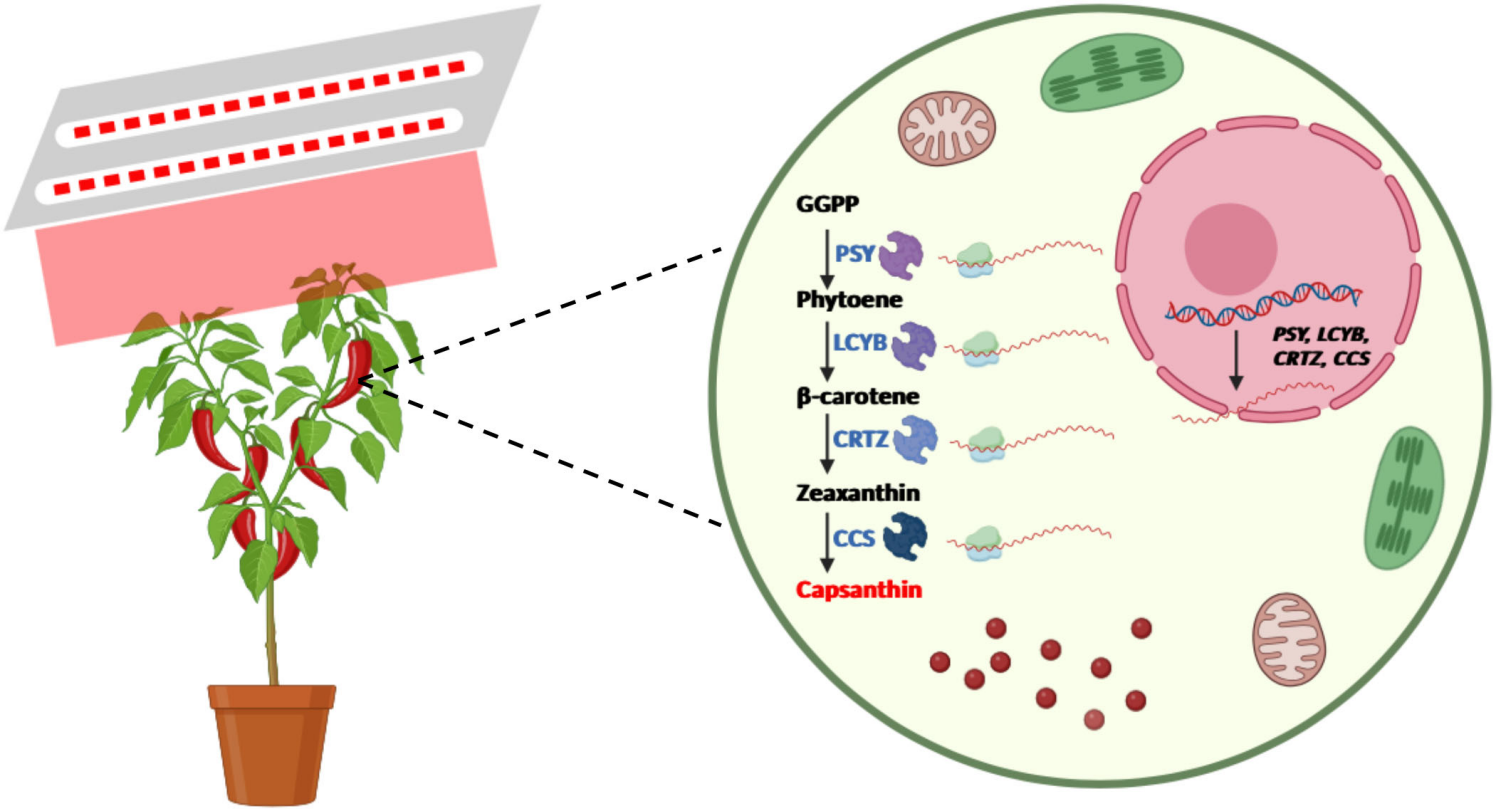
Schematic illustration of the pattern by which pre-harvest red LED light regulates fruit ripening and capsanthin accumulation in pepper (Created in https://BioRender.com).

In summary, the present study investigated the mechanisms by which pre-harvest red LED light regulates capsanthin metabolism in pepper fruits. These results demonstrate that pre-harvest red LED light significantly accelerated color change and enhanced capsanthin content in pepper fruits compared to white LED light. This effect is closely linked to the activation of red light signaling, which upregulates the expression of key genes involved in capsanthin biosynthesis (**Figure 6**). Additionally, VIGS results indicate that the *CCS* gene plays a critical role in red light-mediated color change and capsanthin accumulation in pepper fruits. These findings provide novel insights into how light regulates fruit ripening and offer potential strategies for optimizing the quality of pepper and perhaps other fruits via targeted photobiological interventions.

## Data Availability

The original contributions presented in the study are included in the article/**Supplementary Material**. Further inquiries can be directed to the corresponding authors.
