# Supplementary material for: Red LED light accelerates capsanthin accumulation and fruit ripening in pre-harvest pepper (Capsicum annuum L.)
Source: Front Plant Sci. 2025 Dec 3;16:1680730. doi: 10.3389/fpls.2025.1680730 (PMC12708512; doi:10.3389/fpls.2025.1680730)
Supplement: Supplementary file 1 [file DataSheet1.docx]

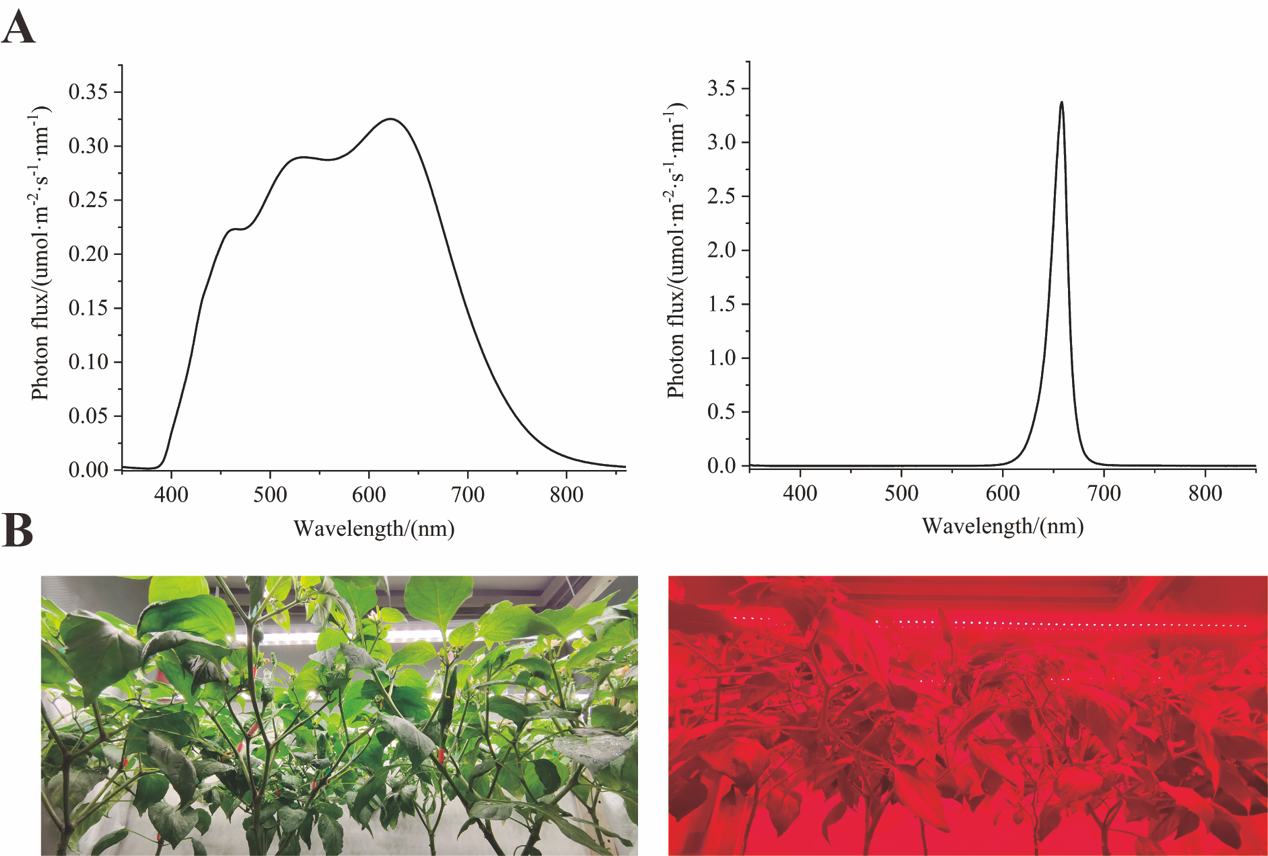


**FIGURE S1** The pepper growing scenario. **(A)** The LED spectrum used in this study. **(B)** Pepper growth environment under the white LED light and red LED light.

**Table S1** The primer sequences of qRT-PCR were used in this study. F: upstream primer; R: downstream primer.

| ID | Gene | Primer | Sequences (5′→3′) |
| --- | --- | --- | --- |
| *Capana04g002519* | *PSY* | *PSY*-F | TCTCAAACGGGACAGGATTC |
|  |  | *PSY*-R | CCACCTTTGTTTTCCACCTC |
| *Capana05g000023* | *LCYB* | *LCYB*-F | AGTTATCCGCAGCTGTTTGG |
|  |  | *LCYB*-R | CCATGCCAATAACGAGGTTC |
| *Capana03g002170* | *CRTZ* | *CRTZ*-F | GCATATGCACGAGTCACACC |
|  |  | *CRTZ*-R | GGGATAAGGCCTTTATGGTT |
| *Capana06g000615* | *CCS* | *CCS*-F | TCGAAAGCCTTGGCTCAACA |
|  |  | *CCS*-R | GAAAGGAACCCGTGCCAGTA |
| *Capana06g002873* | *UBI3* | *UBI3*-F | TGTCCATCTGCTCTCTGTTG |
|  |  | *UBI3*-R | CACCCCAAGCACAATAAGAC |

**Table S2** The Primer sequences of VIGS were used in plasmid construction. F: upstream primer; R: downstream primer. CCTCCATGGGGATCC and CGTGAGCTCGGTACC is a restriction enzyme site of *BamH* I and *Kpn* I, respectively.

| Gene | Primer | Sequences (5′→3′) |
| --- | --- | --- |
| pTRV2/*PSY* | pTRV2/*PSY*-F | CCTCCATGGGGATCC TGCCTTGTTATGGGTTGTTT |
|  | pTRV2/*PSY*-R | CGTGAGCTCGGTACC CCTTCTTCACATCTAACTCATCG |
| pTRV2/*LCYB* | pTRV2/*LCYB*-F | CCTCCATGGGGATCC CCACATGGTTAGCAATCAACTC |
|  | pTRV2/*LCYB*-R | CGTGAGCTCGGTACC CACAGAGCTAAAGGCACTAAC |
| pTRV2/*CRTZ* | pTRV2/*CRTZ*-F | CCTCCATGGGGATCC CAAAATACTTTGCAACTGCCC |
|  | pTRV2/*CRTZ*-R | CGTGAGCTCGGTACC TAATAAACTGAAATAACCGCCAT |
| pTRV2/*CCS* | pTRV2/*CCS*-F | CCTCCATGGGGATCC CCTTTTCCATCTCCTTTACTT |
|  | pTRV2/*CCS*-R | CGTGAGCTCGGTACC CTGTCCAAATACTTAGTCTTGTGAT |
